# Computed tomography-guided percutaneous trephine removal of the nidus in osteoid osteoma patients: experience of a single center in Brazil*

**DOI:** 10.1590/0100-3984.2014.0024

**Published:** 2015

**Authors:** Marcelo Petrilli, Andreza Almeida Senerchia, Antonio Sergio Petrilli, Henrique Manoel Lederman, Reynaldo Jesus Garcia Filho

**Affiliations:** 1 MD, Orthopedic Surgeon, Instituto de Oncologia Pediátrica – GRAACC/Unifesp, São Paulo, SP, Brazil.; 2 MD, Medical Manager of Clinical Research Department, Instituto de Oncologia Pediátrica – GRAACC/Unifesp, São Paulo, SP, Brazil.; 3 PhD, Medical Director, Instituto de Oncologia Pediátrica – GRAACC/Unifesp, São Paulo, SP, Brazil.; 4 PhD, Head of Radiology Department, Instituto de Oncologia Pediátrica – GRAACC/Unifesp, São Paulo, SP, Brazil.; 5 PhD, Head of Orthopedic Department, Instituto de Oncologia Pediátrica – GRAACC/Unifesp, São Paulo, SP, Brazil.

**Keywords:** Osteoma osteoid, Benign tumor, Nidus, CT-guided, Image-guided

## Abstract

**Objective:**

To report the results of computed tomography (CT)-guided percutaneous resection of the nidus in 18 cases of osteoid osteoma.

**Materials and Methods:**

The medical records of 18 cases of osteoid osteoma in children, adolescents and young adults, who underwent CT-guided removal of the nidus between November, 2004 and March, 2009 were reviewed retrospectively for demographic data, lesion site, clinical outcome and complications after procedure.

**Results:**

Clinical follow-up was available for all cases at a median of 29 months (range 6–60 months). No persistence of pre-procedural pain was noted on 17 patients. Only one patient experienced recurrence of symptoms 12 months after percutaneous resection, and was successfully retreated by the same technique, resulting in a secondary success rate of 18/18 (100%).

**Conclusion:**

CT-guided removal or destruction of the nidus is a safe and effective alternative to surgical resection of the osteoid osteoma nidus.

## Abstract (pt)

**Objetivo:**

Avaliar os resultados da ressecção percutânea do nidus guiada por tomografia computadorizada em 18 casos de osteoma osteoide.

**Materiais e Métodos:**

Os prontuários médicos de 18 crianças, adolescentes e adultos jovens diagnosticados com osteoma osteoide e submetidos a ressecção percutânea do nidus guiada por TC, entre novembro de 2004 e março de 2009, foram revisados retrospectivamente para coleta de dados demográficos, local da lesão, evolução clínica e complicações após o procedimento.

**Resultados:**

A mediana de tempo de seguimento de todos os pacientes foi 29 meses (intervalo de 6–60 meses). Dezessete pacientes não apresentaram recorrência da dor no pós-operatório. Apenas um paciente teve recorrência dos sintomas 12 meses após a ressecção percutânea e foi retratado com sucesso, pela mesma técnica, resultando numa taxa de sucesso secundário de 18/18 (100%).

**Conclusão:**

A ressecção guiada por tomografia computadorizada do nidus é uma alternativa segura e eficaz para a ressecção cirúrgica do nidus em pacientes com osteoma osteoide.

## INTRODUCTION

Osteoid osteoma is a benign bone tumor, characterized by a cortically, subperiosteal or intramedullary located nidus with a variable amount of calcification, as well as cortical thickening, sclerosis, and bone marrow edema. Males are affected more than females, at approximately a 2–3:1 ratio, and usually occurs in young individuals (5–25 years)^(1-3)^. 

The bones that are most affected are the femur and the tibia, accounting for 50% of the cases, but almost any bone can be involved^(4)^. It presents with intense and typically nocturnal pain, which can be alleviated by aspirin and cured by removing the nidus^(5)^.

The nidus is round or oval with low attenuation and well defined by computed tomography (CT). Also, an area of high attenuation may be seen centrally, representing mineralized osteoid^(6,7)^.

In the past, open surgery was performed and the nidus had to be removed with a bone block, but is often unsuccessful because the nidus is difficult to find and remove completely^(8)^. CT-guided percutaneous biopsy of bone lesions as a diagnostic tool has shown to be accurate and safe^(9-11)^. In order to achieve removal or destruction of the nidus several CT-guided percutaneous techniques was developed.

The advantages of these methods include the ease of pinpointing the nidus with tomography and a reduced morbidity rate due to minimal resection of the bone mainly by using radio frequency ablation. Its disadvantages include the high cost of acquiring the probes and specific materials required by these techniques^(12-14)^. The technique of CTguided percutaneous resection of the nidus is appealing because it has a low cost because there is no need for special materials and the hospitalization time is short.

We retrospectively evaluated the results of 18 cases of osteoid osteoma treated with percutaneous trephine resection of the nidus under CT guidance at a single institution.

## MATERIALS AND METHODS

We reviewed medical data of 18 consecutive patients treated at the Institute of Pediatric Oncology-GRAACC/Federal University of São Paulo by CT-guided percutaneous trephine resection for osteoid osteoma between November, 2004 and March, 2009. In all, 15 of the patients were male and 3 female. The mean/median age at diagnosis was 18 years (10 to 34 years). The bones affected were the femur in 7 cases, the tibia in 6, and the humerus, ulna, cuneiforme, calcaneus and fibula in 1 each. Five patients were affected by intra-articular osteoid osteoma (4 in the proximal femur and 1 in the proximal ulna).

All patients gave written informed consent after having been explained the treatment procedure, possible complications, and the alternative treatment option of open surgical excision by the orthopedic surgeon. The local ethics committee approved the procedure of CT-guided percutaneous resection.

The osteoid osteoma was diagnosed from clinical and imaging findings. The clinical diagnoses were made based on history and radiographic examinations including plain xray and CT scans. Inclusion criteria were predefined as follows: patients reporting severe pain that usually worsened at night, and were receiving nonsteroidal anti-inflammatory drugs for pain relief at least in the past three months.

**Figure 1:**
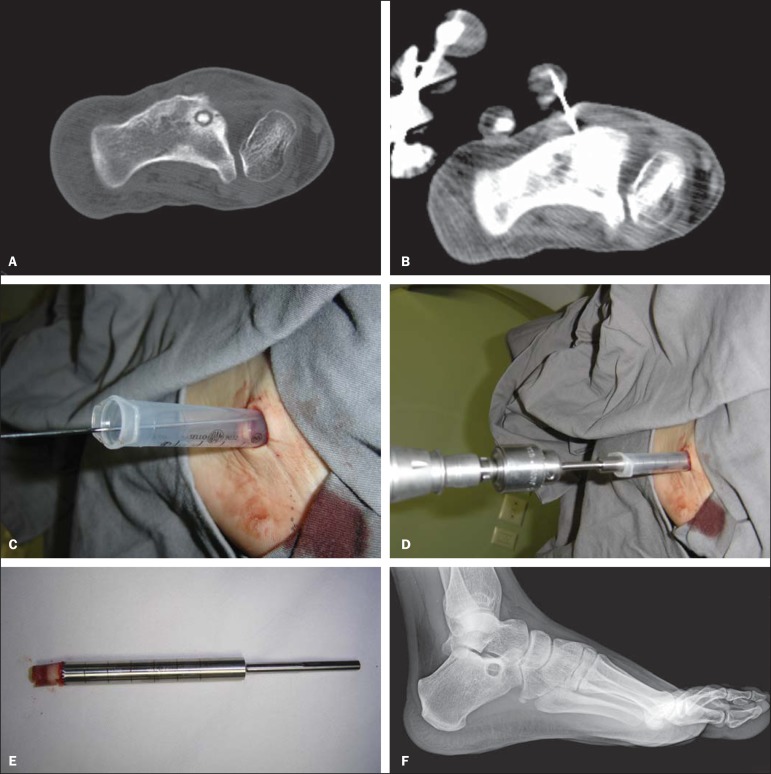
**A:** A well-defined round nidus is visible in the calcaneus CT image. **B:** Needle placed into the nidus to precise location of the guide wire. **C,D:** Cannulated drill is guided over the wire into the nidus. **E:** Nidus removed. **F:** Radiograph after discharged.

**Figure 2:**
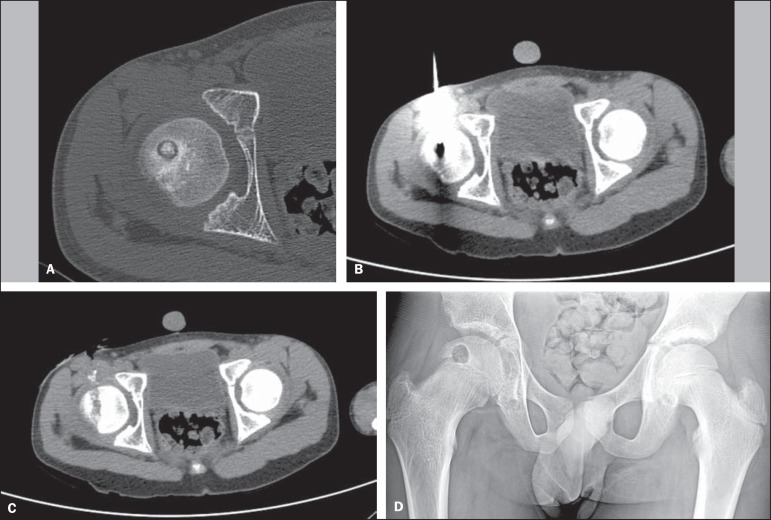
**A:** CT scan shows a radiolucent nidus with central calcification and surrounding reactive sclerosis. **B:** Drilling the cortex of the hip using a cannulated drill over the wire, which is positioned inside the nidus. **C:** Immediate control scan, showing the complete resection of the lesion. **D:** Radiograph after discharged.

The operation was performed under sedation and local anesthesia on an inpatient basis, using the same technique by the same orthopedic surgeon. A X 8000 Dual Philips CT apparatus, with interval cuts of 2 mm, was used. After pinpointing the nidus by tomography, the skin was marked in the area of the lesion. A 0.5 cm incision was made through the skin and a 2.5 mm Kirschner wire was initially driven percutaneously into the nidus under CT guidance. The track up to the nidus was next drilled and enlarged by a 10 mm Corin cannulated drill bit (Arthrex). At this point, a cannulated curette was inserted into the nidus. When in place, it was used to remove the nidus mechanically. The nidus was removed and tissue biopsy was obtained for histological evaluation. CT reconfirmed that the nidus was in fact removed. The cutaneous wound was sutured and the patients were discharged in a period no longer than 12 hours after the procedure (Figures 1 and 2).

The improvement in the clinical symptoms was analyzed, focusing mainly on the improvement regarding pain by using the Musculoskeletal Tumor Society Rating Scale^(14)^.

Follow-up consisted of a repeat clinical evaluation as well as plain x-rays taken at one week, one month, and every three months thereafter. All patients were followed for a period of at least 6 months, with a median follow-up of 29 months (range 6 to 60 months).

Standard statistical descriptive parameters (enumerations, proportions, mean/medians, and ranges) are used to characterize the data.

## RESULTS

All procedures were technically successful. All patients included were discharged in a period no longer than 12 hours after the procedure. One patient had delayed wound healing due to the contact of the trephine with his skin, without any further sequela. Clinical success was achieved in 94.5% of patients (17/18). One patient continued to have some pain in his foot up to 30 months after the procedure with no recurrence of osteoid osteoma.

Clinical follow-up was available for all cases at a median of 29 months (range, 6–60 months), being 12 of all followed for at least 25 months. Only one patient had recurrence of symptoms 12 months after percutaneous resection and was successfully retreated by the same technique, resulting in a secondary success rate of 18/18 (100%). Demographic data are presented in Table 1.

**Table 1:** Patient demographics and clinical course

Case	Age (years)	Gender	Tumor site	Preoperative pain	Postoperative pain	Follow-up period (months)	Recurrence
1	18	Male	Proximal femur	3	5	58	No
2	12	Male	Tibia	3	5	60	No
3	13	Female	Tibia	3	5	50	No
4	13	Male	Tibia	2	5	56	No
5	17	Male	Proximal femur	0	5	46	No
6	26	Female	Tibia	3	5	40	No
7	12	Female	Proximal femur	3	5	39	No
8	20	Male	Humerus	3	5	28	No
9	14	Male	Proximal femur	0	5	28	No
10	12	Male	Tibia	3	5	25	No
11	26	Male	Cuneiform	2	4	30	No
12	22	Male	Fibula	3	5	35	Yes
13	19	Male	Femur	3	5	17	No
14	15	Male	Proximal ulna	4	5	16	No
15	21	Male	Calcaneus	2	5	13	No
16	34	Male	Tibia	3	5	11	No
17	22	Male	Proximal femur	2	5	7	No
18	10	Male	Proximal femur	3	5	6	No

Function and quality of life was assessed pre- and postoperatively using the Musculoskeletal Tumor Society Rating Scale, which is a seven-item scale rated by the clinician and evaluates mainly clinical measures (pain, joint range of motion, strength, joint stability, joint deformity, overall function, and general acceptance of the treatment)^(14)^. The summary score ranges from 0 to 5 in each. The mean preoperative pain score improved from 2.5 (range, 0 to 4) to 4.94 (range, 4 to 5) after procedure.

## DISCUSSION

We presented a successful experience in treating osteiod osteoma in 18 patients with percutaneous trephine resection of the nidus under CT guidance. Success rates of 77–100% have been reported in previous studies using CT-guided percutaneous techniques^(13,15,16)^. The procedure had a primary and secondary success rate of 17/18 (94.5%) and 18/ 18 (100%), respectively, in this study.

Even though rarely cases have been reported even after 44 months^(17)^, relapse is unlikely after 2 years and the standard accepted follow-up period in the orthopedics literature is a minimum of 2 years^(18)^. We followed all patients for a median follow-up of 29 months (range, 6–60 months) and 12 of all were followed for at least 25 months. The one who experienced recurrence, it occurred 12 months after primary treatment.

Limitations of our study include retrospective design and small sample size, however as previously described as advantage of the minimally invasive procedures of nidus removal; diminished hospitalization time and less post-operative pain were seen in our patient population.

Although it has been superseded by the percutaneous ablative techniques such as radiofrequency, CT guided percutaneous resection of the nidus was effective in treating primary as well as recurrent osteoid osteoma in 18 patients. The demographic data were compatible with published literature such as high success rate found with no incidence of complications^(19-21)^.

## CONCLUSION

The aim of treating patients with osteoid osteoma by completely remove the nidus, thereby eliminating symptoms in the young patient population predominantly affected by this tumor was achieved in all patients with low incidence of complications. CT-guided percutaneous trephine resection of the nidus was safely performed with diminished hospitalization time and less post-operative pain.

## References

[1] Iyer RS, Chapman T, Chew FS (2012). Pediatric bone imaging: diagnostic imaging of osteoid
osteoma. AJR Am J Roentgenol.

[2] Chai JW, Hong SH, Choi JY (2010). Radiologic diagnosis of osteoid osteoma: from simple to challenging
findings. Radiographics.

[3] Albisinni U, Rimondi E, Bianchi G (2004). Experience of the Rizzoli Institute in radiofrequency thermal ablation
of musculoskeletal lesions. J Chemother.

[4] Gangi A, Alizadeh H, Wong L (2007). Osteoid osteoma: percutaneous laser ablation and follow-up in 114
patients. Radiology.

[5] Healey JH, Ghelman B (1986). Osteoid osteoma and osteoblastoma. Current concepts and recent
advances. Clin Orthop Relat Res.

[6] Gamba JL, Martinez S, Apple J (1984). Computed tomography of axial skeletal osteoid osteomas. AJR Am J Roentgenol.

[7] Allen SD, Saifuddin A (2003). Imaging of intra-articular osteoid osteoma. Clin Radiol.

[8] Woertler K, Vestring T, Boettner F (2001). Osteoid osteoma: CTguided percutaneous radiofrequency ablation and
follow-up in 47 patients. J Vasc Interv Radiol.

[9] Chojniak R, Grigio HR, Bitencourt AGV (2012). Percutaneous computed tomography-guided core needle biopsy of soft
tissue tumors: results and correlation with surgical specimen
analysis. Radiol Bras.

[10] Cotta AC, Melo RT, Castro RCR (2012). Diagnostic difficulties in osteoid osteoma of the elbow: clinical,
radiological and histopathological study. Radiol Bras.

[11] Maciel MJS, Tyng CJ, Barbosa PNVP (2014). Computed tomography- guided percutaneous biopsy of bone lesions: rate
of diagnostic success and complications. Radiol Bras.

[12] Peyser A, Applbaum Y, Khoury A (2007). Osteoid osteoma: CT-guided radiofrequency ablation using a
water-cooled probe. Ann Surg Oncol.

[13] Rehnitz C, Sprengel SD, Lehner B (2012). CT-guided radiofrequency ablation of osteoid osteoma and
osteoblastoma: clinical success and long-term follow up in 77
patients. Eur J Radiol.

[14] Enneking WF, Enneking WF (1987). Modification of the system for functional evaluation in the surgical
management of musculoskeletal tumors. Limb salvage in musculoskeletal oncology.

[15] Akhlaghpoor S, Tomasian A, Arjmand Shabestari A (2007). Percutaneous osteoid osteoma treatment with combination of
radiofrequency and alcohol ablation. Clin Radiol.

[16] Reverte-Vinaixa MM, Velez R, Alvarez S (2013). Percutaneous computed tomography-guided resection of non-spinal
osteoid osteomas in 54 patients and review of the literature. Arch Orthop Trauma Surg.

[17] Sofka CM, Saboeiro GR, Schneider R (2006). Magnetic resonance imaging diagnosis and computed tomography-guided
radiofrequency ablation of osteoid osteoma. HSS J.

[18] de Berg JC, Pattynama PM, Obermann WR (1995). Percutaneous computed-tomography-guided thermocoagulation for osteoid
osteomas. Lancet.

[19] Yang WT, Chen WM, Wang NH (2007). Surgical treatment for osteoid osteoma - experience in both
conventional open excision and CT-guided mini-incision surgery. J Chin Med Assoc.

[20] Sierre S, Innocenti S, Lipsich J (2006). Percutaneous treatment of osteoid osteoma by CT-guided drilling
resection in pediatric patients. Pediatr Radiol.

[21] Fenichel I, Garniack AQ, Morag B (2006). Percutaneous CT-guided curettage of osteoid osteoma with histological
confirmation: a retrospective study and review of the literature. Int Orthop.

